# Analysis of Reported Aflatoxin Levels in EU’s Rapid Alert System for Food and Feed (RASFF) Notifications

**DOI:** 10.3390/foods14183250

**Published:** 2025-09-19

**Authors:** Ron Kow, Benjamin Er, Joanna Khoo, Joanne Sheot Harn Chan, Kyaw Thu Aung

**Affiliations:** 1 National Centre for Food Science, Singapore Food Agency, 7 International Business Park, Singapore 609919, Singapore; 2 Department of Food Science & Technology, Faculty of Science, National University of Singapore, 2 Science Drive 2, Singapore 117543, Singapore; 3 School of Biological Sciences, Nanyang Technological University, 60 Nanyang Drive, Singapore 637551, Singapore

**Keywords:** aflatoxins, mycotoxins, RASFF notifications, food recalls

## Abstract

Aflatoxins are one of the major mycotoxins contaminating food and feed. This study analyses 14 years of notifications on aflatoxins (2010–2023) from the European Union’s Rapid Alert System for Food and Feed (RASFF) to assess contamination risk based on notification counts and the reported aflatoxin levels. The distribution of food and feed in notifications indicates that peanuts (34.4%), pistachios (17.3%) and figs (12.5%) have the highest contamination risk. Among major food–origin combinations, significant differences in the distributions of aflatoxin levels suggest the influence of country-specific factors on contamination patterns. The data also revealed a distinct seasonal peak in notifications on figs from Türkiye in the last quarter of the year, as well as rising trends in median aflatoxin levels over 14 years for figs from Türkiye and pistachios from Iran, with each having a fivefold increase in median levels from 2010 to 2023.

## 1. Introduction

Mycotoxins are the toxic secondary metabolites of fungi [1]. Aflatoxins are a group of 15 to 20 closely related mycotoxins produced by various species of fungi in the genus *Aspergillus*. The two major species responsible for aflatoxin production in food crops are *A. flavus* and *A. parasiticus* [2]. Aflatoxins are carcinogens known to target the liver and are associated with many serious health conditions [3]. The most common forms of aflatoxins are aflatoxin B_1_, B_2_, G_1_ and G_2_ (denoted as AFB_1_, AFB_2_, AFG_1_ and AFG_2_, respectively, in this paper). As seen on a chromatographic plate under ultraviolet light, the abbreviations B and G indicate the colours blue and green, and the numbers 1 and 2 indicate the relative migration distance of the compounds [2]. Global food recall data indicate that aflatoxins B and G are found in a wide variety of food and animal feed, especially peanuts, tree nuts, dried fruits, cereal grains, seeds, peppers and spices.

Aflatoxin M_1_ (AFM_1_), a metabolic product of AFB_1_, is found in animal tissues and fluids. When lactating humans and animals feed on food and feed contaminated with AFB_1_, AFB_1_ may potentially be converted to AFM_1_ and excreted in the milk. The conversion of AFB_1_ to AFM_1_ is influenced by factors such as genetics, the milking process and environmental conditions [4]. Although there are far fewer recalls globally of food products contaminated with AFM_1_ compared to recalls due to AFB_1_, AFM_1_ in dairy products is a particular concern because infants and young children consume milk daily. It is also known that other forms of aflatoxins, including AFB_1_ and AFG_1_, can contaminate dairy products (and human milk) [5]. The International Agency for Research on Cancer (IARC) classifies both AFB_1_ and AFM_1_ as Group 1 carcinogens [4].

Examining global food recall records provides valuable insights into how aflatoxins contaminate food supply chains worldwide. The European Union’s Rapid Alert System for Food and Feed (RASFF) provides one of the most comprehensive collections of data on food recalls in the world. Launched in 1979, RASFF enables fast exchange of information on health risks related to food and feed among member countries [6]. Other than countries in the EU, RASFF member countries include countries from the European Economic Area (Iceland, Liechtenstein and Norway), Switzerland and the European Food Safety Authority (EFSA). The number of yearly notifications posted by RASFF has grown from less than 500 notifications in 2000 to more than 4600 notifications in 2023.

Much research on RASFF data has been published in recent years, including analyses of notifications on mycotoxins in specific food [7] and notifications on aflatoxins in food from specific countries of origin [8]. However, prior research on RASFF notifications has largely overlooked the reported aflatoxin levels. By analysing 14 years of RASFF notifications on aflatoxins (2010–2023), the objectives of this study are to identify the food and feed with the highest contamination risk, compare the distributions of the reported aflatoxin levels across major food–origin combinations, and discover temporal trends in notification counts and median aflatoxin levels of food–origins.

## 2. Materials and Methods

### 2.1. RASFF Notification Data

Every RASFF notification from 2020 to 2023 has an individual web page that can be viewed on the internet and is searchable from a web portal called RASFF Window [9]. The data are also available for download in Excel and CSV formats from RASFF Window. The downloaded dataset contains a subset of information shown on the web page of each notification. As data on the reported aflatoxin levels are not found in the dataset, we scraped the web page of each notification on aflatoxins to obtain the data.

Pre-2020 notifications, except the few which have been updated since 2020, are not searchable from RASFF Window and are not available for viewing on the internet. Older data from 1979 to 2019 are available for download from the EU’s European Data Portal [10]. Similar to the 2020–2023 dataset, this pre-2020 dataset contains a subset of the complete information in each notification. However, it includes data on the reported aflatoxin levels.

### 2.2. Data Processing and Analysis

The reported levels required careful cleaning to ensure accuracy. Units of measurement were removed, and commas used in decimal values were replaced by decimal points. In many notifications, multiple levels were reported, indicating multiple samples collected or multiple lab tests performed. Due to recording errors and the inconsistent format of the reported results, it was not clear in a small number of notifications if a reported value referred to AFB_1_ or the sum of AFB_1_, AFB_2_, AFG_1_ and AFG_2_. For cases reported in 2020–2023 notifications, we manually checked the web page of each notification. If there was no clear indication on the web page, we assumed the value to be the AFB_1_ level. Most reported levels also indicate the uncertainty in measurement (e.g., 8.9 ± 2.3). These were all removed. Notifications with ambiguously recorded levels were excluded from this study.

Some notifications had multiple countries of origin. These notifications were on food or feed processed or packaged in one country using raw commodities produced in another country. Although contamination may occur during processing or packaging, we took the origin to be the country of production of the raw commodity.

All data processing and analysis were performed using programs written in Python version 3.8.8. Medians were calculated using the median function in NumPy version 1.20.1. The Kruskal–Wallis H test [11] and the Mann–Whitney U test [12] from SciPy version 1.6.2 were used for tests of differences in the distributions of aflatoxin levels of food origins. Significance levels were adjusted using the Bonferroni correction where appropriate. All charts were plotted using Microsoft Power BI version 2.129.905.0.

## 3. Results

Before studying the reported aflatoxin levels, it is informative to investigate the prevalence of aflatoxin contamination in food and feed. The distribution of food and feed in notifications on aflatoxins from 2010 to 2023 provides an estimation of the risk of aflatoxin contamination in food or feed commodities. For dairy products, we present the reported AFM_1_ levels in notifications from 2010 to 2023. For other food products, we present and analyse the reported AFB_1_, AFB_2_, AFG_1_ and AFG_2_ levels in notifications from 2020 to 2023.

### 3.1. RASFF Notifications by Mycotoxin Type (2010–2023)

The EU has established maximum limits for six major mycotoxins (aflatoxins, ochratoxin A, deoxynivalenol, fumonisins, patulin and zearalenone) and their associated food commodities [13]. Many of the food commodities in notifications on aflatoxins have established maximum limits. Table 1 shows the number of notifications by mycotoxin type from 2010 to 2023.

There were 7277 notifications on mycotoxins, of which 6442 (88.5%) concerned aflatoxins. In 79 notifications on aflatoxins, other mycotoxins were also detected. Aflatoxins and ochratoxin A were both reported in 71 notifications, contaminating fruits (dates, figs, etc.), nuts (peanuts, pistachios), cereal grains (corn, rice), spices (chili peppers, nutmegs, paprika, etc.) and melon seeds. Aflatoxins and fumonisins were both reported in six notifications on corn. Aflatoxins and zearalenone were both reported in one notification on rice. Aflatoxins and tenuazonic acid were both reported in one notification on dried figs.

Other mycotoxins (other than aflatoxins and ochratoxin A) were detected in a small basket of food types. Deoxynivalenol was detected in cereal grains (corn, wheat, etc.); fumonisins were detected in corn and peanuts; zearalenone was detected in rice and wheat; patulin was detected in apple juice and paste; and alternaria mycotoxins (alternariol, tenuazonic acid) were detected in tomato products.

### 3.2. Distribution of Food and Feed in RASFF Notifications on Aflatoxins (2010–2023)

**Figure 1:**
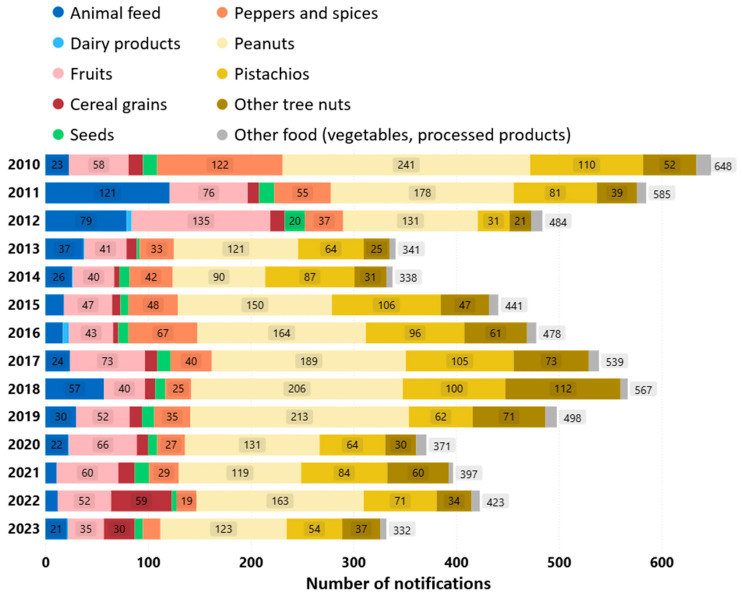
Yearly number of notifications by food and feed commodity type (2010–2023). Data used with permission from Refs. [9,10]. 2025, European Commission.

Figure 1 shows the yearly number of notifications in ten food and feed categories from 2010 to 2023: animal feed, dairy products, fruits, cereal grains, seeds, peppers and spices, peanuts, pistachios, other tree nuts and other food. Pistachios are presented separately from other tree nuts due to the large number of notifications. The last category (“other food”) contains mainly processed products made from multiple raw commodities. We observe rising trends (2014–2018, 2020–2022) and falling trends (2010–2013, 2018–2020) in the yearly number of notifications.

Table 2 shows the number of notifications for food and feed in two separate periods (2010–2019 and 2020–2023). From 2010 to 2023, there were a total of 6442 notifications on aflatoxins, of which 498 notifications were on animal feed. The two most common raw materials in animal feed were peanuts (384 notifications) and corn (59). Other feed materials were sunflower seeds (19), rice (7) and a variety of other commodities (29), such as millet, sorghum, soybeans, cottonseeds and coconuts.

The top three food commodity types were peanuts (2219), tree nuts (1808) and fruits (818 notifications, of which 805 notifications were on figs). Peanuts and peanut products had the most notifications every year. There were 70 notifications on peanut butter and peanut paste, and 14 notifications on peanut powder and peanut flour.

Aflatoxins contaminated nine different tree nuts, but 95.5% of the notifications on tree nuts concerned pistachios, almonds and hazelnuts. Pistachio was the leading tree nut, with 1115 notifications. The number of notifications on other tree nuts varied significantly, led by hazelnuts (406), almonds (206) and Brazil nuts (31). The other types of tree nuts (chestnuts, cashew nuts, walnuts, pecans and pine nuts) had less than 10 notifications each. There were 17 notifications on mixed or unspecified nuts.

RASFF data indicate that dried fruits such as dried figs, dried dates and raisins are particularly vulnerable to aflatoxin contamination. Of the 805 notifications on figs, 783 notifications were specified as dried figs, and 13 notifications were on fig paste. Other than figs, the only other types of fruits were dates (10) and mulberries (3). Seven notifications on dates concerned processed dates (dried, pitted, diced or in syrup form).

The data indicate that aflatoxins contaminate a wide range of peppers and spices. Notifications on peppers were predominantly on chili peppers (257). A total of 42 notifications on peppers were specified as dried peppers, and 183 notifications were on peppers in ground or crushed form. A total of 93 notifications on spices were specified as mixed spices. These include common spice mixes such as curry and kebab spices. For individual spice types, there were many notifications on nutmegs (151) and a smaller number of notifications on ginger (31), turmeric (20), paprika (16), black and white peppercorns (3) and cumin (2).

The data indicate that cereal grains are at risk from most mycotoxins. For aflatoxins, rice and corn (including rice flour and corn flour) were the most common cereal grains, with 158 and 45 notifications, respectively. Other cereal grains were millet (6), buckwheat (4), wheat (2), barley (1) and spelt (1).

Eight types of oilseeds and fruit seeds were found in notifications on aflatoxins, led by melon seeds, with 98 notifications. Other seeds were apricot kernels (16), sesame seeds (10), ogbono seeds (9), sunflower seeds (8), chia seeds (7), lotus seeds (2) and flaxseeds (1).

Lastly, there were 120 notifications that we classified as “other food”. These concerned food that does not belong to any of the other nine categories, such as beans and tiger nuts, and prepared or processed food such as sauces, seasonings, snacks and food for infants. Many of these food products contain peanuts, tree nuts, fruits or cereal grains as ingredients.

### 3.3. AFM_1_ Levels in Dairy Products (2010–2023)

There were 15 notifications on AFM_1_ in dairy products from 2010 to 2023: milk (10 notifications), cheese (four) and whey (one) (Table 3). Notably, eight of the notifications on milk were on raw milk. All products originated from Europe (Hungary, Italy, Serbia and Slovenia). The reported levels in 14 notifications on milk and cheese ranged from 0.063 µg/kg to 0.87 µg/kg, all exceeding the EU’s regulatory maximum levels of 0.05 µg/kg (for raw milk, heat-treated milk and milk used for the manufacture of milk-based products) and 0.025 µg/kg (for formula milk) [13].

### 3.4. AFB_1_, AFB_2_, AFG_1_, AFG_2_ Levels in Food Commodities (2020–2023)

Considering that the countries of origin of some food commodities imported into Europe may have changed significantly since 2010, the notifications in the most recent years provide more relevant information for assessing current origin-specific risks. We therefore focused our analysis on the levels reported in notifications from 2020 to 2023.

Due to differences in sample sizes (i.e., the number of levels reported) for different food commodities and the presence of extremely high levels in a few samples, we present the median levels (rather than mean levels) for all food commodities. Table 4 presents the median, minimum and maximum levels of AFB_1_ and the sum of AFB_1_, AFB_2_, AFG_1_ and AFG_2_ for each food commodity or product.

### 3.5. AFB_1_, AFB_2_, AFG_1_, AFG_2_ Levels in Food Commodities by Country of Origin (2020–2023)

As the number of notifications and reported levels were very low for many food commodities, we focused on six major food commodities (figs, rice, peanuts, pistachios, hazelnuts and almonds) and their major countries of origins. Table 5 presents the country-specific aflatoxin levels of the six major food commodities. Each food commodity had at least 50 notifications, and each country of origin had at least 10 notifications from 2020 to 2023. Box plots showing the distributions of aflatoxin levels of the food–origins in Table 5 are presented in Figure S1.

Tests of differences in the distributions of aflatoxin levels of the food–origins indicate significant differences. The results of the Kruskal–Wallis H tests for peanuts, pistachios and hazelnuts (each with more than two major countries of origins) are presented in Tables S1 and S2. The results of the Mann–Whitney U tests for pairwise comparisons are presented in Tables S3 and S4. Non-transitive inconsistencies in the test results are possibly due to unequal sample sizes and outliers.

### 3.6. Trends in AFB_1_, AFB_2_, AFG_1_, AFG_2_ Levels of Food-Origins (2010–2023)

**Figure 2:**
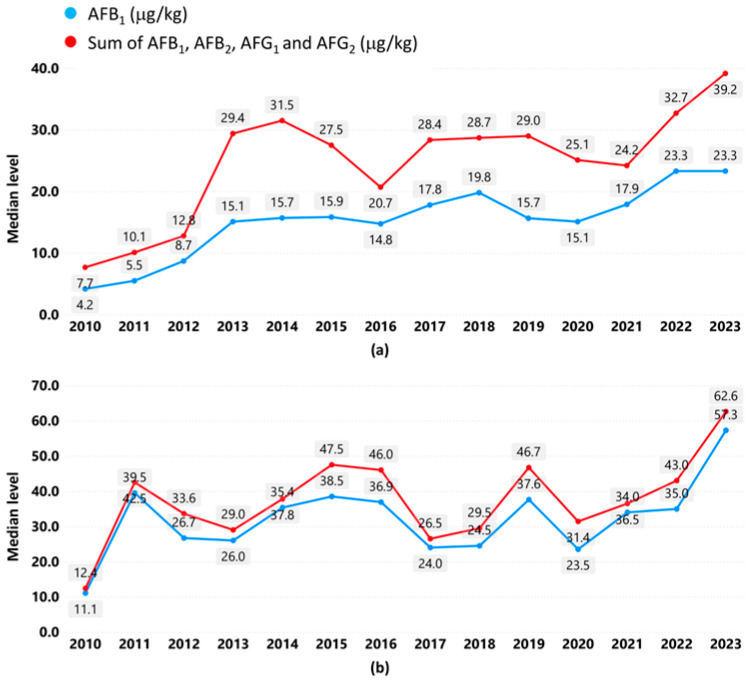
(**a**) Yearly median levels for figs from Türkiye (2010–2023); (**b**) yearly median levels for pistachios from Iran (2010–2023). Data used with permission from Refs. [9,10]. 2025, European Commission.

We plotted the yearly median levels from 2010 to 2023 for the food–origins in Table 5 to study long-term trends in aflatoxin levels. The plots for figs from Türkiye and pistachios from Iran show rising trends over 14 years (Figure 2). For both food–origins, there were fivefold increases in median levels from 2010 to 2023.

For figs from Türkiye (Figure 2a), the median AFB_1_ increased from 4.2 µg/kg in 2010 (sample size *n* = 83) to 23.3 µg/kg in 2023 (*n* = 25), while the median sum of AFB_1_, AFB_2_, AFG_1_ and AFG_2_ increased from 7.7 µg/kg in 2010 (*n* = 76) to 39.2 µg/kg in 2023 (*n* = 30).

For pistachios from Iran (Figure 2b), there were sharp increases in median levels from 2010 to 2011 and from 2022 to 2023, resulting in an increase in the median AFB_1_ from 11.1 µg/kg in 2010 (*n* = 114) to 57.3 µg/kg in 2023 (*n* = 23) and in an increase in the median sum of AFB_1_, AFB_2_, AFG_1_ and AFG_2_ from 12.4 µg/kg in 2010 (*n* = 113) to 62.6 µg/kg in 2023 (*n* = 23).

## 4. Discussion

### 4.1. Factors Influencing Aflatoxin Production

Aflatoxin production in food and feed is influenced by many factors in the environment of the fungi. Factors shown to enhance or reduce aflatoxin production include the availability of water (which is essential for both fungal growth and aflatoxin production), temperature, radiation, the nutrients in the crop, the presence of bacteria and the presence of many chemical compounds, such as organic solvents and pesticides [2]. Many of these factors affect all stages of the food chain. For instance, dried fruits are at risk during pre-harvest, from the moment the fruit tree bears fruit to post-harvest, when fruits are processed, transported and stored. Likely factors influencing aflatoxin production in dried fruits include the additional time spent in drying and processing the fruits after harvest and the likelihood of longer storage before consumption [14].

Various studies over recent decades [15] have indicated that climate is a factor in aflatoxin production. The Food and Agriculture Organization (FAO) of the United Nations and the European Food Safety Authority (EFSA) have identified aflatoxins as one of the foodborne hazards most likely to be impacted by climate change [16,17].

### 4.2. Correlation Between Import Quantity and Number of Notifications of Food–Origins

Import quantity tends to correlate with the number of notifications. For example, Türkiye is the largest source of figs for RASFF member countries, accounting for 71.6% of dried fig imports from 2010 to 2023 [18]. This correlated with the large number of notifications on figs from Türkiye (96.1% of notifications on figs from 2010 to 2023). In the last two decades, the import of pistachios from Iran to RASFF member countries fell from 54.1% of total pistachio imports in 2000 to 4.6% in 2023 [18]. Iran accounted for most of the notifications on pistachios before 2009, with over 400 notifications per year from 2003 to 2005. Europe’s reduced dependence on Iran for pistachios correlated with a sharp decrease in notifications on pistachios from Iran after 2005. This reduced the overall number of notifications on pistachios from more than 500 notifications in 2003 to fewer than 100 notifications in most of the years since 2011.

However, a disproportionately large number of notifications on some food–origins suggests underlying country-specific factors influencing aflatoxin contamination. For example, Pakistan was the fourth largest source of rice, accounting for 9.5% of rice imports from 2020 to 2023 [18]. However, there were more notifications on rice from Pakistan (66) than from all other countries of origin combined (25).

### 4.3. Seasonal Trends in Number of Notifications of Food–Origins

The RASFF data from 2010 to 2023 showed a distinct seasonal trend in the number of notifications on figs from Türkiye (Figure S2), with spikes in notifications during the fourth quarter of most of the years. The plot of the distribution of notification counts by month over 14 years shows low notification counts from February to September, followed by sharp increases from October to January (Figure S3a). This trend may be associated with the yearly growing season of figs, which depends on climate. For Turkish figs, the main yearly harvest occurs around August. Aydin Province in western Türkiye is the dominant region for the production of dried figs [19]. A lag between harvesting and the dates of notifications is expected due to the time required for processing, exporting, sampling and testing of dried figs. We also observed decreasing trends, followed by increases in notifications in the last quarter of the year for pistachios and hazelnuts from Türkiye (Figure S3b,c). For hazelnuts from Georgia (Figure S3d) and Azerbaijan (Figure S3e), where the climate is similar to that of Türkiye, notification counts were also very low during summer months before increasing in the final four months of the year. The similar trends observed for Türkiye, Georgia and Azerbaijan suggest an association between climate and notification counts. The summer conditions in July and August may have an influence on aflatoxin contamination in crops, leading to higher detection rates in the final three to four months of the year.

### 4.4. Aflatoxin Levels of Food–Origins (2020–2023)

The significant differences in aflatoxin levels between some food–origins suggest that contamination levels may be influenced by country-specific factors. Although climate and other natural environmental factors may be influential, man-made factors related to farming practices and regulatory controls can also have an impact. For example, as the climate of Türkiye and Azerbaijan is quite similar, the significantly higher levels reported for hazelnuts from Azerbaijan could be due to man-made country-specific factors.

Figure 2 shows a rising trend and a fivefold increase in yearly median levels over 14 years for figs from Türkiye and pistachios from Iran. This may be an indication of the increasing risk to the safety of figs and pistachios from Türkiye and Iran, respectively. The EU reviews its food safety regulations for specific country–food–hazards at regular intervals (not exceeding six months) based on RASFF data and other information. It implements temporary changes of official controls and emergency measures for countries showing increasing non-compliance. The measures include increasing the frequency of checks and sampling, requirement of an official certificate from the country of origin proving compliance by stating results of sampling, and suspension of entry into the EU [20]. The EU has implemented these measures for dried figs from Türkiye and pistachios from Iran since 2019 [20,21]. The observed rising trends suggest that the measures have not produced the desired effect.

## 5. Conclusions

Historical RASFF notifications provide quantitative evidence for identifying high-risk food products and prevalent contaminants. Studying long-term trends in RASFF notifications for specific food–origins helps the EU in evidence-based risk assessment and management of food–origins, informing decisions on import controls. The distribution of food and feed in notifications from 2010 to 2023 indicates that certain food commodities (peanuts, pistachios, hazelnuts, almonds, figs, rice, melon seeds, peppers and nutmegs) have a higher risk of aflatoxin contamination. Peanuts, pistachios and figs, with 34.4%, 17.3% and 12.5% of all notifications, respectively, have the highest risk. Significant differences in the distributions of aflatoxin levels between food–origins suggest country-specific factors influencing aflatoxin contamination. RASFF data also revealed a distinct seasonal trend in notifications on figs from Türkiye, with yearly spikes in notifications in the last quarter of most years. A fivefold increase in median levels over 14 years for figs from Türkiye and pistachios from Iran are a concern for the EU, as both countries are major import sources.

Monitoring trends in RASFF notifications and learning from EU’s risk management measures may help countries outside of Europe assess aflatoxin risk and make informed decisions on import sources, particularly small countries that may not be able to conduct enough testing if import quantities are much smaller.

## Figures and Tables

**Table 1:** Number of notifications on mycotoxins (2010–2023). Data used with permission from Refs. [9,10]. 2025, European Commission.

Mycotoxin	Number of Notifications
Aflatoxins	6363
Ochratoxin A	674
Aflatoxins and Ochratoxin A	71
Deoxynivalenol	60
Fumonisins	48
Patulin	20
Zearalenone	8
Aflatoxins and Fumonisins	6
Deoxynivalenol and Zearalenone	6
Ochratoxin A and Deoxynivalenol	2
Aflatoxins and Tenuazonic Acid	1
Aflatoxins and Zearalenone	1
Deoxynivalenol and Fumonisins	1
Other mycotoxins ^1^	16
Total	7277

^1^ Citrinin, Lolitrem B, Alternariol, Tenuazonic Acid, T-2, HT-2.

**Table 2:** Number of aflatoxin notifications for food and feed (2010–2023). Data used with permission from Refs. [9,10]. 2025, European Commission.

Category	Commodity or Product	Number of Notifications 2010–2019	Number of Notifications 2020–2023	Total
Animal feed	Peanut feed	343	41	384
	Corn feed	49	10	59
	Sunflower feed	15	4	19
	Rice feed	4	3	7
	Others (compound feed, etc.)	21	8	29
Dairy products	Cheese	3	1	4
	Milk, other products	10	1	11
Fruits	Figs	603	202	805
	Dates	2	8	10
	Mulberries	0	3	3
Cereal grains	Rice	67	91	158
	Corn	30	15	45
	Millet	2	4	6
	Buckwheat	1	3	4
	Wheat	0	2	2
	Barley	1	0	1
	Spelt	0	1	1
Seeds	Melon seeds	77	21	98
	Apricot kernels	14	2	16
	Sesame seeds	3	7	10
	Ogbono seeds	7	2	9
	Sunflower seeds	7	1	8
	Chia seeds	6	1	7
	Lotus seeds	1	1	2
	Flaxseeds	0	1	1
Peppers and spices	Peppers	247	33	280
	Nutmegs	123	28	151
	Ginger	27	4	31
	Turmeric	13	7	20
	Paprika	15	1	16
	Peppercorns (black, white)	1	2	3
	Cumin	2	0	2
	Mixed spices	76	17	93
Peanuts and tree nuts	Peanuts	1683	536	2219
	Pistachios	842	273	1115
	Hazelnuts	315	91	406
	Almonds	149	57	206
	Brazil nuts	24	7	31
	Chestnuts	8	1	9
	Cashew nuts	5	2	7
	Walnuts	6	1	7
	Pecans	5	0	5
	Pine nuts	5	0	5
	Mixed nuts or unspecified	15	2	17
Other food	Processed products	85	27	112
	Vegetables	7	1	8
Total	-	4919	1523	6442

**Table 3:** AFM_1_ levels in dairy products (2010–2023). Data used with permission from Refs. [9,10]. 2025, European Commission.

Product	Number of Notifications	AFM_1_ (µg/kg)			*n* ^1^	Remarks
		Median	Min	Max		
Raw milk	8	0.10	0.063	0.214	6	No AFM_1_ results were reported in 5 notifications.
Milk	2	0.15	0.152	0.152	1	
Cheese	4	0.24	0.07	0.87	6	
Whey	1	-	-	-	0	
Total	15	**-**	**-**	**-**	13	

^1^ *n* is the sample size, i.e., the number of levels reported in the notifications.

**Table 4:** AFB_1_, AFB_2_, AFG_1_, AFG_2_ levels in food commodities (2020–2023). Data used with permission from Refs. [9,10]. 2025, European Commission.

Category	Commodity or Product	AFB_1_ (µg/kg)				Sum of AFB_1_, AFB_2_, AFG_1_ and AFG_2_ (µg/kg)			
		Median	Min	Max	*n* ^1^	Median	Min	Max	*n* ^1^
Fruits	Figs	18.0	1.0	822.0	188	27.0	0.49	540.0	186
	Dates	3.85	2.6	70.0	12	62.0	12.0	73.0	3
	Mulberries	6.6	6.2	8.3	3	9.35	9.35	9.35	1
Cereal grains	Rice	6.9	0.21	48.0	102	13.5	1.4	122.5	42
	Corn	7.8	2.74	44.0	19	9.45	5.1	49.0	12
	Millet	6.95	5.7	8.2	4	8.4	6.2	9.5	4
	Buckwheat	15.78	4.3	32.7	4	17.0	6.1	38.0	4
	Wheat	8.6	8.6	8.6	1	8.6	8.6	8.6	1
	Barley	-	-	-	0	-	-	-	0
	Spelt	3.7	3.7	3.7	1	11.3	11.3	11.3	1
Seeds	Melon seeds	10.2	3.3	79.3	16	13.05	7.9	88.2	16
	Apricot kernels	58.0	9.4	80.5	4	77.35	17.7	110.0	4
	Sesame seeds	6.7	3.9	38.0	7	12.2	6.7	15.3	4
	Ogbono seeds	12.15	9.3	15.0	2	20.85	9.7	32.0	2
	Sunflower seeds	3.5	3.5	3.5	1	9.3	9.3	9.3	1
	Chia seeds	6.1	4.3	7.9	2	10.0	10.0	10.0	1
	Lotus seeds	12.0	12.0	12.0	1	-	-	-	0
	Flaxseeds	5.9	4.1	7.7	2	7.8	5.1	10.5	2
Peppers and spices	Peppers	12.6	6.9	299.0	33	20.25	10.0	356.2	14
	Nutmegs	22.0	7.09	170.0	33	26.8	8.6	220.0	27
	Ginger	12.8	6.7	31.0	4	31.25	14.8	38.4	4
	Turmeric	10.7	9.2	15.5	7	14.9	14.9	14.9	1
	Paprika	8.3	8.3	8.3	1	-	-	-	0
	Peppercorns (black, white)	12.1	9.5	14.7	2	20.1	20.1	20.1	1
	Cumin	-	-	-	0	-	-	-	0
	Mixed spices	10.0	6.6	26.8	15	18.0	12.6	78.8	7
Peanuts and tree nuts	Peanuts	12.0	0.5	407.0	631	22.0	0.6	480.0	499
	Pistachios	29.9	1.7	1064.0	289	34.8	3.1	1170.0	286
	Hazelnuts	17.4	2.8	489.2	84	29.5	2.8	562.0	85
	Almonds	16.8	0.2	420.0	71	23.1	2.0	466.0	67
	Brazil nuts	15.0	7.7	80.3	6	27.5	11.1	167.0	6
	Chestnuts	4.3	4.3	4.3	1	7.7	7.7	7.7	1
	Cashew nuts	31.7	31.7	31.7	1	41.75	9.6	73.9	2
	Walnuts	31.85	3.9	59.8	2	45.1	5.1	85.1	2
	Pecans	-	-	-	0	-	-	-	0
	Pine nuts	-	-	-	0	-	-	-	0
	Mixed nuts or unspecified	32.2	32.2	32.2	1	36.1	36.1	36.1	1
Other food	Processed products	13.87	0.28	139.0	26	15.4	4.3	158.0	19
	Vegetables	7.8	7.8	7.8	1	13.2	13.2	13.2	1

^1^ *n* is the sample size, i.e., the number of levels reported in the notifications.

**Table 5:** AFB_1_, AFB_2_, AFG_1_, AFG_2_ levels of major food–origins (2020–2023). Data used with permission from Refs. [9,10]. 2025, European Commission.

Commodity	Country of Origin	No. of Notifications	AFB_1_ (µg/kg)				Sum of AFB_1_, AFB_2_, AFG_1_ and AFG_2_ (µg/kg)			
			Median	Min	Max	*n* ^1^	Median	Min	Max	*n* ^1^
Figs	Türkiye	192	17.8	1.0	822.0	178	27.5	0.49	540.0	176
Rice	Pakistan	66	7.2	0.5	48.0	73	13.5	1.4	122.5	38
	India	10	4.2	3.0	24.0	11	27.0	27.0	27.0	1
Peanuts	USA	128	11.6	0.5	407.0	136	20.0	3.2	480.0	103
	Egypt	115	25.5	3.32	320.0	146	34.4	0.6	370.0	133
	Argentina	100	6.45	2.1	110.0	116	13.0	2.4	140.0	85
	India	89	11.5	2.82	260.0	104	22.0	4.5	270.0	73
	China	25	12.2	2.4	107.7	29	24.0	4.3	122.9	25
	Bolivia	19	23.0	4.1	300.0	28	59.55	8.6	350.0	22
Pistachios	Iran	111	35.55	9.7	1064.0	116	42.36	13.0	1170.0	114
	Türkiye	74	25.04	1.7	225.0	75	27.5	3.1	309.0	76
	USA	68	29.7	7.9	240.0	78	33.05	8.7	260.0	78
Hazelnuts	Georgia	57	18.02	5.9	489.2	50	31.6	12.2	562.0	53
	Azerbaijan	21	28.5	6.9	320.0	22	44.25	11.01	370.0	22
	Türkiye	12	8.3	2.8	46.0	11	20.3	2.8	51.0	9
Almonds	USA	25	16.8	0.2	277.0	29	24.0	13.2	302.0	24
	Australia	17	14.7	0.5	50.0	29	21.4	2.0	125.0	25

^1^ *n* is the sample size, i.e., the number of levels reported in the notifications.

## Data Availability

The 2020–2023 RASFF data used in this study are available for download from the RASFF website (https://webgate.ec.europa.eu/rasff-window/screen/search (accessed on 22 May 2025)). Older 2000–2019 data are available for download from the EU’s European Data Portal (https://data.europa.eu/en (accessed on 16 February 2024)). Food trade data are available from the FAOSTAT Food and Agriculture Data website (https://www.fao.org/faostat/en/ (accessed on 26 May 2025)).

## Supplementary Materials

The following supporting information can be downloaded at https://www.mdpi.com/article/10.3390/foods14183250/s1. Table S1: Kruskal–Wallis H test results for comparisons of the distributions of AFB_1_ levels across food–origins (2020–2023); Table S2: Kruskal–Wallis H test results for comparisons of the distributions of sums of the AFB_1_, AFB_2_, AFG_1_ and AFG_2_ levels across food–origins (2020–2023); Table S3: Mann–Whitney U test results for pairwise comparisons of the distributions of AFB_1_ levels across food–origins (2020–2023); Table S4: Mann–Whitney U test results for pairwise comparisons of the distributions of sums of the AFB_1_, AFB_2_, AFG_1_ and AFG_2_ levels across food–origins (2020–2023); Figure S1: Distributions of aflatoxin levels across food–origins (2020–2023); Figure S2: Quarterly notification counts of figs from Türkiye (2010–2023); Figure S3: Distributions of monthly notification counts by food–origin (2010–2023).

## Abbreviations

The following abbreviations are used in this manuscript:

RASFF	Rapid Alert System for Food and Feed
EU	European Union
EC	European Commission
AFM_1_	Aflatoxin M_1_
AFB_1_	Aflatoxin B_1_
AFB_2_	Aflatoxin B_2_
AFG_1_	Aflatoxin G_1_
AFG_2_	Aflatoxin G_2_

## References

[1] Richard J.L. (2007). Some major mycotoxins and their mycotoxicosis—An overview. Int. J. Food Microbiol..

[2] Klich M.A. (2007). Environmental and developmental factors influencing aflatoxin production by Aspergillus flavus and Aspergillus parasiticus. Mycoscience.

[3] Gong Y.Y., Watson S., Routledge M.N. (2016). Aflatoxin exposure and associated human health effects, a review of epidemiological studies. Food Saf..

[4] Iqbal S.Z., Jinap S., Pirouz A.A., Faizal A.A. (2015). Aflatoxin M1 in milk and dairy products, occurrence and recent challenges: A review. Trends Food Sci. Technol..

[5] Galvano F., Galofaro V., Galvano G. (1996). Occurrence and Stability of Aflatoxin M1 in Milk and Milk Products: A Worldwide Review. J. Food Prot..

[6] Rapid Alert System for Food and Feed (RASFF). https://food.ec.europa.eu/food-safety/rasff_en.

[7] Owolabi I.O., Karoonuthaisiri N., Elliot C.T., Petchkongkaew A. (2023). A 10-year analysis of RASFF notifications for mycotoxins in nuts. Trend in key mycotoxins and impacted countries. Food Res. Int..

[8] Alshannaq A., Yu J.-H. (2021). Analysis of E.U. Rapid Alert System (RASFF) Notifications for Aflatoxins in Exported U.S. Food and Feed Products for 2010–2019. Toxins.

[9] European Commission (2025). RASFF Window. https://webgate.ec.europa.eu/rasff-window/screen/search.

[10] European Commission (2025). European Data Portal. https://data.europa.eu/en.

[11] Kruskal W.H., Wallis W.A. (1952). Use of ranks in one-criterion variance analysis. J. Am. Stat. Assoc..

[12] Mann H.B., Whitney D.R. (1947). On a test of whether one of two random variables is stochastically larger than the other. Ann. Math. Stat..

[13] Commission Regulation (EU) 2023/915 of 25 April 2023 on Maximum Levels for Certain Contaminants in Food and Repealing Regulation (EC) No 1881/2006. https://eur-lex.europa.eu/legal-content/EN/TXT/?uri=CELEX%3A02023R0915-20250101.

[14] González-Curbelo M.Á., Kabak B. (2023). Occurrence of Mycotoxins in Dried Fruits Worldwide, with a Focus on Aflatoxins and Ochratoxin A: A Review. Toxins.

[15] Cotty P.J., Jaime-Garcia R. (2007). Influences of climate on aflatoxin producing fungi and aflatoxin contamination. Int. J. Food Microbiol..

[16] Food and Agriculture Organization of the United Nations (FAO) (2020). Climate change: Unpacking the Burden on Food Safety. Food Safety and Quality Series No. 8.

[17] Maggiore A., Afonso A., Barrucci F., Sanctis G.D., European Food Safety Authority (EFSA) (2020). Climate change as a driver of emerging risks for food and feed safety, plant, animal health and nutritional quality. EFSA Support. Publ..

[18] FAOSTAT https://www.fao.org/faostat/en/.

[19] Uzundumlu A.S., Oksuz M.E., Kurtoglu S. (2018). Future of Fig Production in Turkey. J. Tekirdag Agric. Fac..

[20] Commission Implementing Regulation (EU) 2019/1793 of 22 October 2019 on the Temporary Increase of Official Controls and Emergency Measures Governing the Entry into the Union of Certain Goods from Certain Third Countries Implementing Regulations (EU) 2017/625 and (EC) No 178/2002 of the European Parliament and of the Council and Repealing Commission Regulations (EC) No 669/2009, (EU) No 884/2014, (EU) 2015/175, (EU) 2017/186 and (EU) 2018/1660. https://eur-lex.europa.eu/eli/reg_impl/2019/1793/oj/eng.

[21] EU Audit Questions Turkish Approach to Control Mycotoxins. https://www.foodsafetynews.com/2023/01/eu-audit-questions-turkish-approach-to-control-mycotoxins/.

