# Supplementary material for: Analysis of Reported Aflatoxin Levels in EU’s Rapid Alert System for Food and Feed (RASFF) Notifications
Source: Foods. 2025 Sep 19;14(18):3250. doi: 10.3390/foods14183250 (PMC12469425; doi:10.3390/foods14183250)
Supplement: Supplementary file 1 [file foods-14-03250-s001.zip › foods-3833211-supplementary.pdf]

foods
MDPI

Supplementary Information

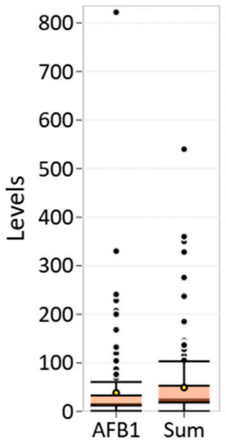

(a) figs-Türkiye

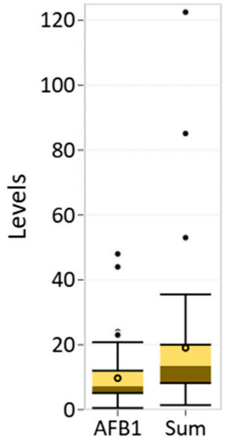

(b) rice-Pakistan

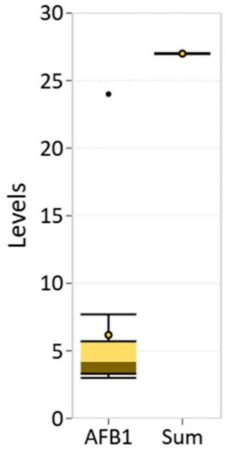

(c) rice-India

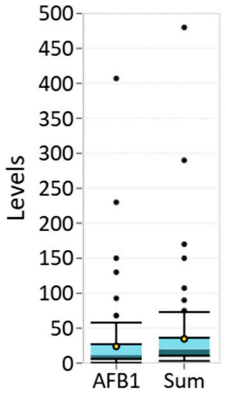

(d) peanuts-USA

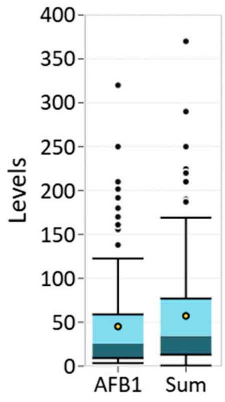

(e) peanuts-Egypt

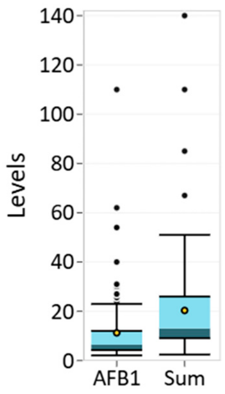

(f) peanuts-Argentina

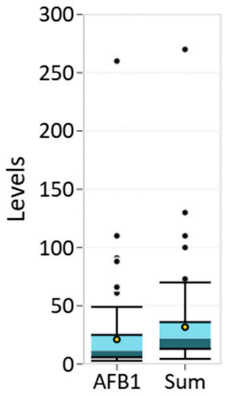

(g) peanuts-India

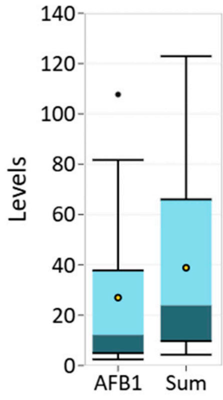

(h) peanuts-China

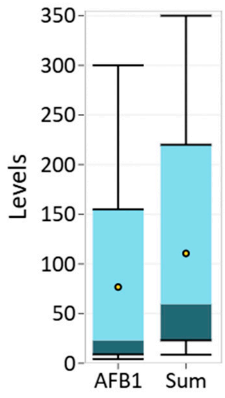

(i) peanuts-Bolivia

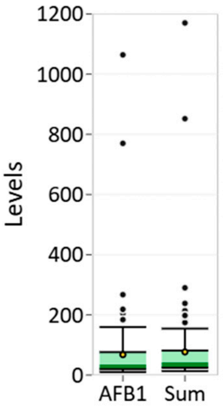

(j) pistachios-Iran

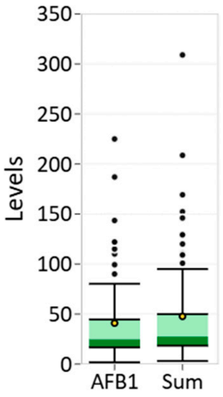

(k) pistachios-Türkiye

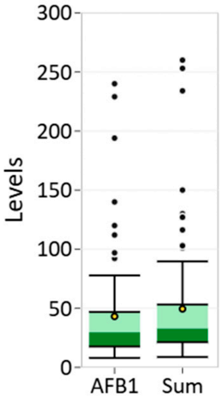

(l) pistachios-USA

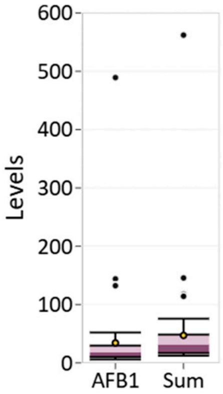

(m) hazelnuts-Georgia

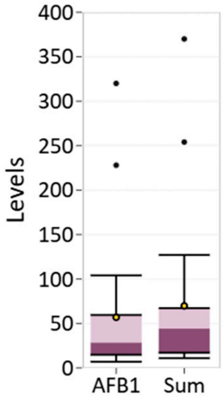

(n) hazelnuts-Azerbaijan

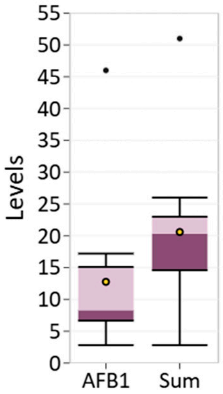

(o) hazelnuts-Türkiye

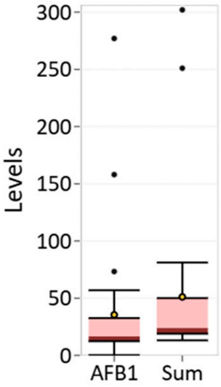

(p) almonds-USA

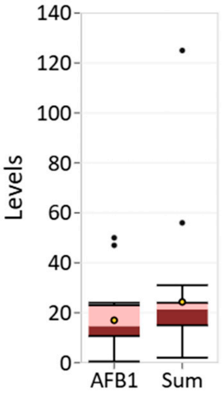

(q) almonds-Australia

Figure S1. Distributions of aflatoxin levels across food–origins (2020–2023). The yellow dot in each box plot indicates the mean level. Data used with permission from Refs. [9,10]. 2025, European Commission.

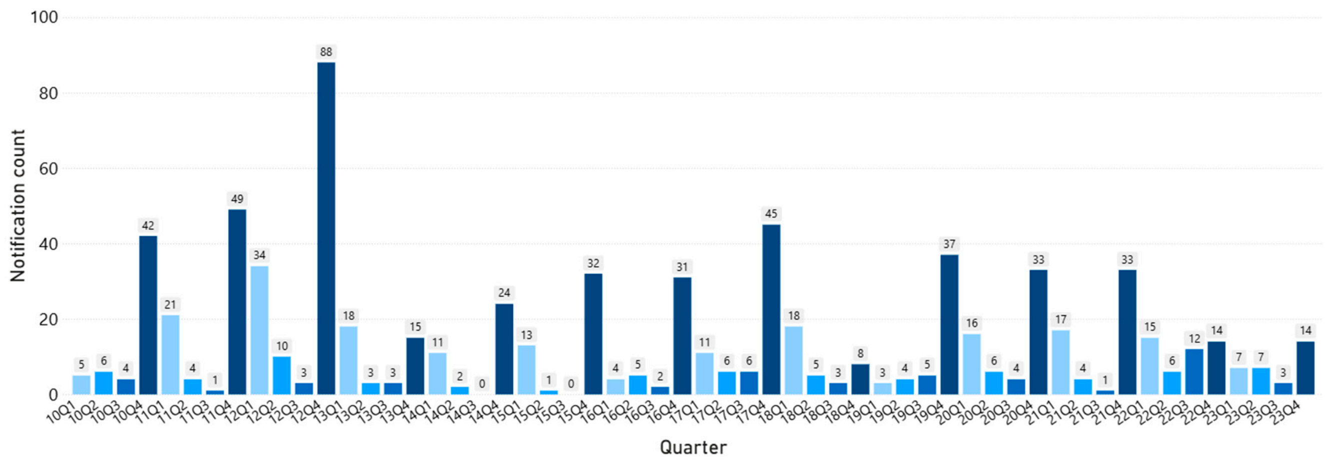

Figure S2. Quarterly notification counts of figs from Türkiye (2010–2023). Data used with permission from Refs. [9,10]. 2025, European Commission.

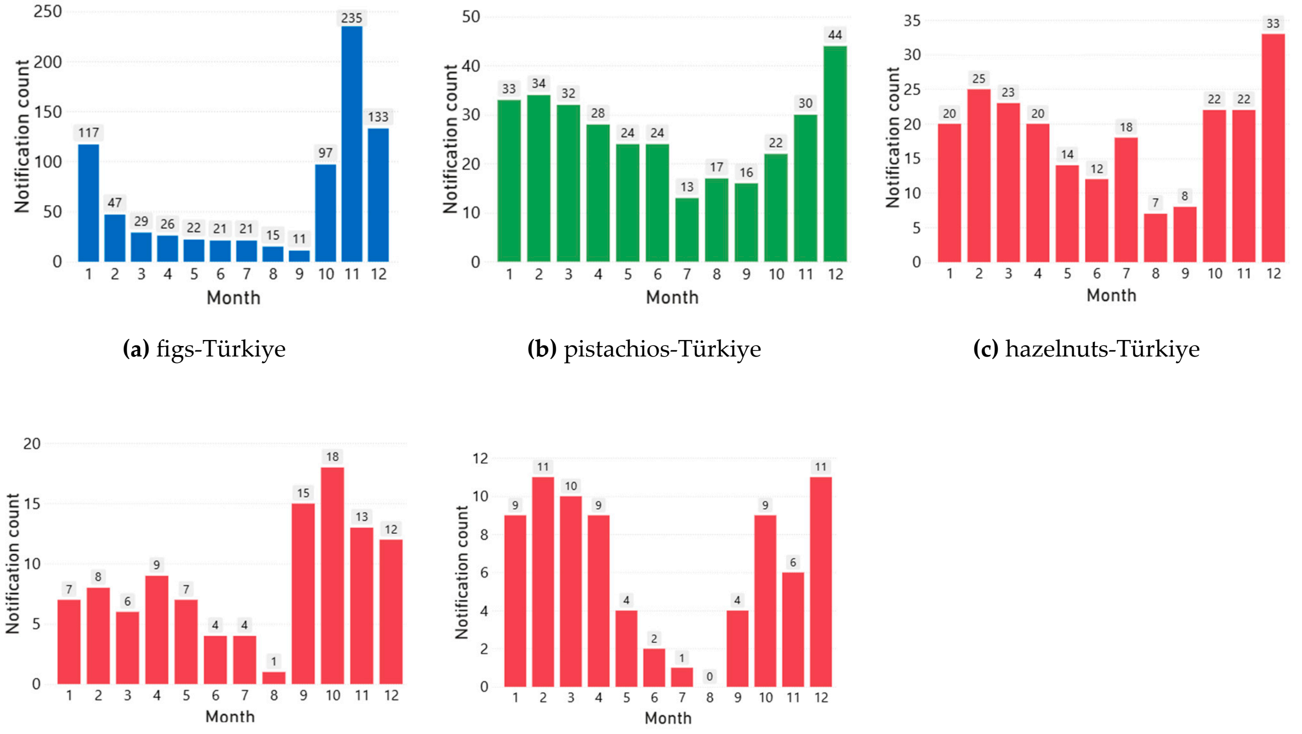

(a) figs-Türkiye **(b)** pistachios-Türkiye **(c)** hazelnuts-Türkiye

(d) hazelnuts-Georgia **(e)** hazelnuts-Azerbaijan

Figure S3. Distributions of monthly notification counts by food–origin (2010–2023). Data used with permission from Refs. [9,10]. 2025, European Commission.

Table S1. Kruskal-Wallis H test results for comparisons of the distributions of AFB_1 levels across food–origins (2020–2023). Data used with permission from Refs. [9,10]. 2025, European Commission.

Food commodity	Countries of origin	H Statistic	*p*-value	Interpretation
Peanuts	USA, Egypt, Argentina, India, China, Bolivia	82.0	< 0.05	The distribution of at least one country was significantly different.
Pistachios	Iran, Türkiye, USA	6.1	< 0.05	The distribution of at least one country was significantly different.
Hazelnuts	Georgia, Azerbaijan, Türkiye	13.0	< 0.05	The distribution of at least one country was significantly different.

Table S2. Kruskal-Wallis H test results for comparisons of the distributions of sums of the AFB_1, AFB_2, AFG_1 and AFG_2 levels across food–origins (2020–2023). Data used with permission from Refs. [9,10]. 2025, European Commission.

Food commodity	Countries of origin	H Statistic	*p*-value	Interpretation
Peanuts	USA, Egypt, Argentina, India, China, Bolivia	46.7	< 0.05	The distribution of at least one country was significantly different.
Pistachios	Iran, Türkiye, USA	10.2	< 0.05	The distribution of at least one country was significantly different.
Hazelnuts	Georgia, Azerbaijan, Türkiye	6.29	< 0.05	The distribution of at least one country was significantly different.

Table S3. Mann-Whitney U test results for pairwise comparisons of the distributions of AFB_1 levels across food–origins (2020–2023). Data used with permission from Refs. [9,10]. 2025, European Commission.

Food commodity	Countries of origin [1]	U Statistic	*p*-value [2]	Interpretation
Rice	Pakistan, India	589.5	< 0.05	The distribution of Pakistan has significantly higher levels than that of India.
Peanuts	Egypt, USA	6680.5	< 0.0033	The distribution of Egypt has significantly higher levels than that of USA.
	USA, Argentina	10381.5	< 0.0033	The distribution of USA has significantly higher levels than that of Argentina.
	USA, India	7098.5	> 0.0033	The distributions of USA and India were not significantly different.
	China, USA	1947.5	> 0.0033	The distributions of China and USA were not significantly different.
	Bolivia, USA	1223.5	< 0.0033	The distribution of Bolivia has significantly higher levels than that of USA.
	Egypt, Argentina	13481.5	< 0.0033	The distribution of Egypt has significantly higher levels than that of Argentina.
	Egypt, India	10188.0	< 0.0033	The distribution of Egypt has significantly higher levels than that of India.
	Egypt, China	2696.5	> 0.0033	The distributions of Egypt and China were not significantly different.
	Egypt, Bolivia	1840.5	> 0.0033	The distributions of Egypt and Bolivia were not significantly different.
	India, Argentina	4068.5	< 0.0033	The distribution of India has significantly higher levels than that of Argentina.
	China, Argentina	1238.5	> 0.0033	The distributions of China and Argentina were not significantly different.
	Bolivia, Argentina	668.5	< 0.0033	The distribution of Bolivia has significantly higher levels than that of Argentina.
	China, India	1503.5	> 0.0033	The distributions of China and India were not significantly different.
	Bolivia, India	923.0	< 0.0033	The distribution of Bolivia has significantly higher levels than that of India.
	Bolivia, China	265.0	> 0.0033	The distributions of Bolivia and China were not significantly different.
Pistachios	Iran, Türkiye	5210.5	< 0.0167	The distribution of Iran has significantly higher levels than that of Türkiye.
	Iran, USA	5111.0	> 0.0167	The distributions of Iran and USA were not significantly different.
	USA, Türkiye	2635.0	> 0.0167	The distributions of USA and Türkiye were not significantly different.
Hazelnuts	Azerbaijan, Georgia	716.0	> 0.0167	The distributions of Azerbaijan and Georgia were not significantly different.
	Azerbaijan, Türkiye	203.0	< 0.0167	The distribution of Azerbaijan has significantly higher levels than that of Türkiye.
	Georgia, Türkiye	421.0	< 0.0167	The distribution of Georgia has significantly higher levels than that of Türkiye.
Almonds	USA, Australia	517.5	> 0.05	The distributions of USA and Australia were not significantly different.

[1] In each pair, the first country has a higher median level than the second country.

[2] The significance level is adjusted for peanuts, pistachios and hazelnuts using the Bonferroni correction.

Table S4. Mann-Whitney U test results for pairwise comparisons of the distributions of sums of the AFB_1, AFB_2, AFG_1 and AFG_2 levels across food–origins (2020–2023). Data used with permission from Refs. [9,10]. 2025, European Commission.

Food commodity	Countries of origin [1]	U Statistic	*p*-value [2]	Interpretation
Rice	Pakistan, India [3]	-	-	-
Peanuts	Egypt, USA	4926.5	< 0.0033	The distribution of Egypt has significantly higher levels than that of USA.
	USA, Argentina	5310.5	> 0.0033	The distributions of USA and Argentina were not significantly different.
	India, USA	3474.5	> 0.0033	The distributions of India and USA were not significantly different.
	China, USA	1162.0	> 0.0033	The distributions of China and USA were not significantly different.
	Bolivia, USA	605.0	< 0.0033	The distribution of Bolivia has significantly higher levels than that of USA.
	Egypt, Argentina	8197.0	< 0.0033	The distribution of Egypt has significantly higher levels than that of Argentina.
	Egypt, India	5977.5	< 0.0033	The distribution of Egypt has significantly higher levels than that of India.
	Egypt, China	1934.0	> 0.0033	The distributions of Egypt and China were not significantly different.
	Bolivia, Egypt	1110.5	> 0.0033	The distributions of Bolivia and Egypt were not significantly different.
	India, Argentina	2141.5	< 0.0033	The distribution of India has significantly higher levels than that of Argentina.
	China, Argentina	781.0	> 0.0033	The distributions of China and Argentina were not significantly different.
	Bolivia, Argentina	365.0	< 0.0033	The distribution of Bolivia has significantly higher levels than that of Argentina.
	China, India	886.5	> 0.0033	The distributions of China and India were not significantly different.
	Bolivia, India	456.5	< 0.0033	The distribution of Bolivia has significantly higher levels than that of India.
	Bolivia, China	171.5	> 0.0033	The distributions of Bolivia and China were not significantly different.
Pistachios	Iran, Türkiye	5444.0	< 0.0167	The distribution of Iran has significantly higher levels than that of Türkiye.
	Iran, USA	5203.0	> 0.0167	The distributions of Iran and USA were not significantly different.
	USA, Türkiye	2596.0	> 0.0167	The distributions of USA and Türkiye were not significantly different.
Hazelnuts	Azerbaijan, Georgia	684.0	> 0.0167	The distributions of Azerbaijan and Georgia were not significantly different.
	Azerbaijan, Türkiye	149.0	< 0.0167	The distribution of Azerbaijan has significantly higher levels than that of Türkiye.
	Georgia, Türkiye	344.5	> 0.0167	The distributions of Georgia and Türkiye were not significantly different.
Almonds	USA, Australia	402.0	< 0.05	The distribution of USA has significantly higher levels than that of Australia.

[1] In each pair, the first country has a higher median level than the second country.

[2] The significance level is adjusted for peanuts, pistachios and hazelnuts using the Bonferroni correction.

[3] Not tested because the sample size of India is 1.
